# Identification of protective postexposure mycobacterial vaccine antigens using an immunosuppression-based reactivation model in the zebrafish

**DOI:** 10.1242/dmm.033175

**Published:** 2018-03-01

**Authors:** Henna Myllymäki, Mirja Niskanen, Hanna Luukinen, Mataleena Parikka, Mika Rämet

**Affiliations:** 1 BioMediTech Institute and Faculty of Medical and Life Sciences, University of Tampere, Tampere FI-33014, Finland; 2 Oral and Maxillofacial Unit, Tampere University Hospital, Tampere FI-33521, Finland; 3 Department of Pediatrics, Tampere University Hospital, Tampere FI-33521, Finland; 4 Department of Children and Adolescents, Oulu University Hospital, Oulu FI-90220, Finland; 5 PEDEGO Research Unit, and, Medical Research Center, University of Oulu, Oulu FI-90014, Finland

**Keywords:** Tuberculosis, Reactivation, Zebrafish, Vaccine antigens

## Abstract

Roughly one third of the human population carries a latent *Mycobacterium tuberculosis* infection, with a 5-10% lifetime risk of reactivation to active tuberculosis and further spreading the disease. The mechanisms leading to the reactivation of a latent *Mycobacterium tuberculosis* infection are insufficiently understood. Here, we used a natural fish pathogen, *Mycobacterium marinum*, to model the reactivation of a mycobacterial infection in the adult zebrafish (*Danio rerio*). A low-dose intraperitoneal injection (∼40 colony-forming units) led to a latent infection, with mycobacteria found in well-organized granulomas surrounded by a thick layer of fibrous tissue. A latent infection could be reactivated by oral dexamethasone treatment, which led to disruption of the granuloma structures and dissemination of bacteria. This was associated with the depletion of lymphocytes, especially CD4^+^ T cells. Using this model, we verified that ethambutol is effective against an active disease but not a latent infection. In addition, we screened 15 mycobacterial antigens as postexposure DNA vaccines, of which RpfB and MMAR_4207 reduced bacterial burdens upon reactivation, as did the Ag85-ESAT-6 combination. In conclusion, the adult zebrafish-*M. marinum* infection model provides a feasible tool for examining the mechanisms of reactivation in mycobacterial infections, and for screening vaccine and drug candidates.

This article has an associated First Person interview with the first author of the paper.

## Abstract (precis)

**Summary:** We characterize the reactivation of a latent mycobacterial infection and identify DNA-based vaccine candidates against the reactivation of tuberculosis using the adult zebrafish *Mycobacterium marinum* infection model.

## INTRODUCTION

Tuberculosis (TB) remains one of the major global health problems. *Mycobacterium tuberculosis* (Mtb), the causative agent of TB, led to 1.4 million deaths and 10.4 million new infections in 2015 (WHO, 2017). The World Health Organization (WHO) estimates that one third of the human population carries a latent TB infection, and therefore has up to a 10% lifetime risk of it reactivating into an active disease. Both vaccines and antibiotics have their limitations in combating TB. Curative antibiotic treatments are lengthy and further complicated by the emergence of multidrug-resistant Mtb strains (WHO, 2017). The only available TB vaccine, Bacillus Calmette Guérin (BCG), is still widely used. Although the BCG vaccine can protect infants from disseminated TB, its ability to induce long-term cell-mediated immune responses is limited, and therefore it does not properly prevent the reactivation of a latent TB infection (Tang et al., 2016). To reach the ambitious goal of eliminating TB by the year 2050, innovative approaches are needed.

As nonhuman primates are the only animal models that fully replicate all phases seen in human TB, it has been challenging to study this area, especially the latency and reactivation of mycobacterial infections (Capuano et al., 2003; Myllymäki et al., 2015). It is known that reactivation of a latent TB infection is often associated with immunosuppression, such as human immunodeficiency virus (HIV), chemotherapy or immunosuppressive drugs (Ai et al., 2016), but the more detailed mechanisms of reactivation remain largely elusive on both the bacterial and host side (Dutta and Karakousis, 2014; Peddireddy et al., 2017). Considering the number of latent TB carriers, preventing the reactivation of latent TB would be a key step in the battle against TB (Matteelli et al., 2017).

During the past couple of decades, the zebrafish (*Danio rerio*) has proven an applicable alternative for modeling TB (Myllymäki et al., 2016). For this purpose, a natural fish pathogen and a close relative of Mtb, *Mycobacterium marinum*, is used (Stinear et al., 2008). Depending on the infectious dose, a *M. marinum* infection in adult zebrafish can lead either to an active or to a naturally latent form of the disease (Parikka et al., 2012; Swaim et al., 2006). In the latent form of human TB, the bacteria are contained in structures termed granulomas, which are surrounded by immune cells and a fibrotic capsule. Although granulomas were long thought to be a protection method elicited solely by the host, it has been found that they also promote persistence of the bacteria (O'Garra et al., 2013; Davis and Ramakrishnan, 2009). Upon a *M. marinum* infection, both adult zebrafish and larvae form granulomas that are highly similar in structure to those in humans (Parikka et al., 2012; Davis et al., 2002), and the immune responses they elicit against mycobacteria share similarities to those in humans (Parikka et al., 2012; Swaim et al., 2006; Hammarén et al., 2014; Clay et al., 2008; Torraca et al., 2015; Pagán et al., 2015; Yang et al., 2012). This has facilitated the translation of some of the results from fish studies to humans (Tobin et al., 2010; Adams et al., 2014; Berg et al., 2016; Thuong et al., 2017).

In the zebrafish, a latent mycobacterial infection can spontaneously reactivate into an active disease (Hammarén et al., 2014). In the current study, we have developed an experimental method to reactivate a latent mycobacterial infection in the adult zebrafish by feeding them the glucocorticoid dexamethasone. We use this model for the characterization of the cellular and molecular mechanisms associated with the reactivation of a mycobacterial infection. In addition, we show that the model can be used in screening for antibiotics and novel vaccine candidates against the reactivation of a latent mycobacterial infection.

## RESULTS

### Dexamethasone treatment leads to an elevated bacterial burden in zebrafish with a latent *M. marinum* infection

**Fig. 1.**
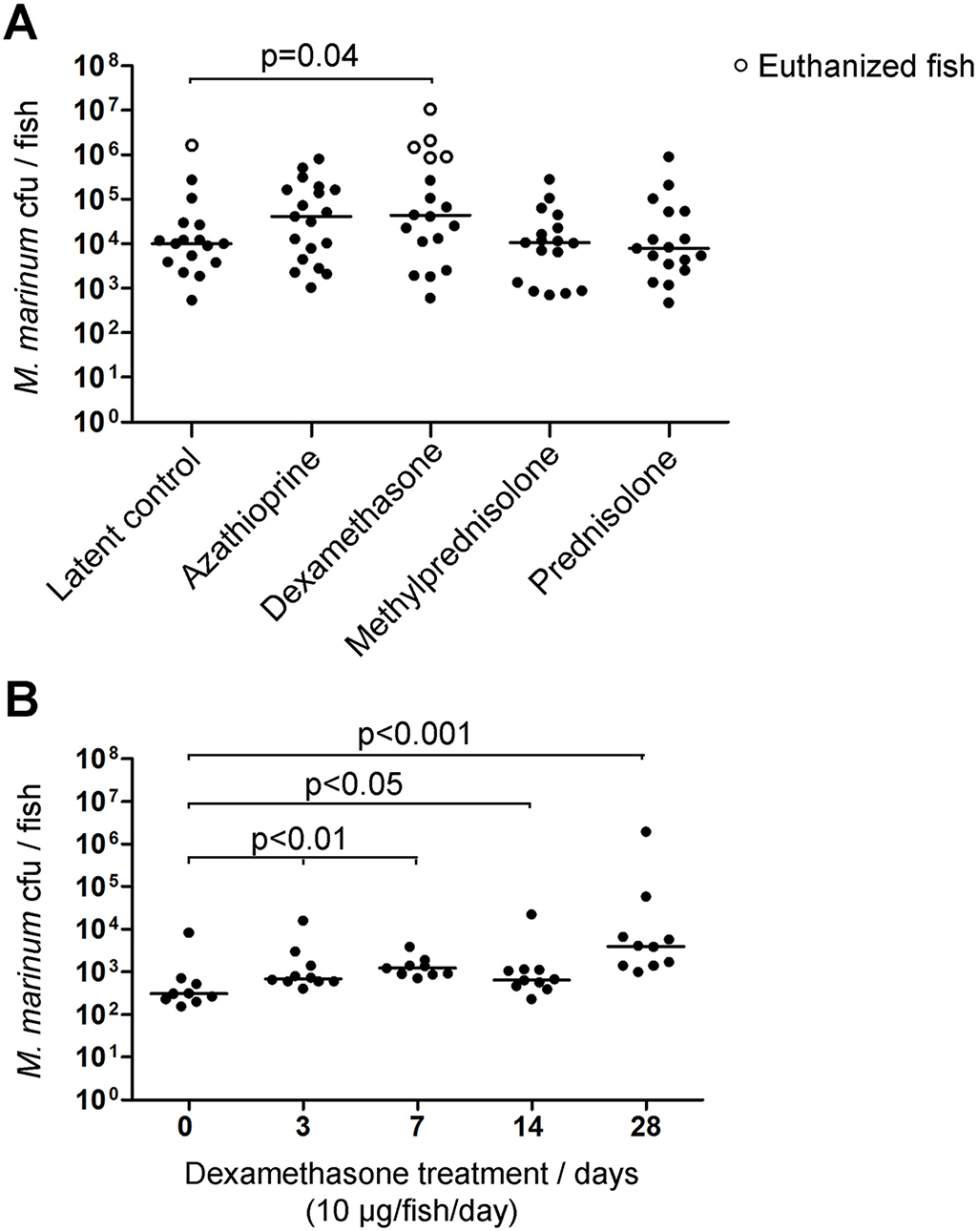
**Dexamethasone treatment increases bacterial loads in adult zebrafish with a latent *M. marinum* infection.** (A) Zebrafish with a latent mycobacterial infection were treated with immunosuppressants. Each dot shows the bacterial count in one fish after 4 weeks of exposure to immunosuppressive medication (10 µg/fish/day), (*n*=17-19 fish/group). (B) Dots represent bacterial loads after 3, 7, 4 and 28 days of dexamethasone treatment of fish with a latent mycobacterial infection (*n*=9-10 fish/group). Horizontal lines represent the median bacterial count of each group. Statistical significance was analyzed with the one-tailed Mann–Whitney test. See also Fig. S1.

In humans, the reactivation of a latent TB infection often follows immunosuppression. Similarly, immunosuppression by gamma irradiation leads to the reactivation of a mycobacterial infection in zebrafish (Parikka et al., 2012). To further study the mechanisms of the reactivation of a mycobacterial infection in zebrafish, we tested the effects of various immunosuppressive medicines on a latent *M. marinum* infection (Fig. 1A). The fish were first infected with a low dose of *M. marinum*, which leads to a latent infection in most fish, and the immunosuppressive treatments were started 5 weeks later. For this, we selected chemicals that are commonly used in humans to alleviate excessive immune reactions associated with various medical situations, such as organ transplantation, arthritis and inflammatory bowel disease, and have been reported to increase risk of the reactivation of TB in individuals with a latent infection (Jick et al., 2006; Maltzman and Koretzky, 2003). The drugs included azathioprine, dexamethasone, methylprednisolone and prednisolone. To minimize additional stress, the drugs were administered orally, as a gelatin mix that was used to coat the fish food pellets. The experimental fish were treated with the drugs, 10 µg/fish/day, for 4 weeks, whereas the control fish received gelatin-coated food with no chemicals. After the treatment, the fish were euthanized and the DNA extracted from the intraperitoneal cavity of each fish was used to determine the number of mycobacteria.

We found that feeding with dexamethasone led to an increase in bacterial burdens (*P*=0.04, one-tailed Mann–Whitney test); azathioprine produced a similar trend, though the increase was not statistically significant (Fig. 1A). Within the anticipated dose of 10 µg/day, prednisolone and methylprednisolone did not lead to elevated bacterial burdens. One fish from the control group and five fish from the group fed with dexamethasone were euthanized according to the humane endpoint criteria during the treatment – these fish were also included in the analysis. Consistently, a high number of mycobacteria [average of 2.9×10^6^±4.8×10^5^ colony-forming units (cfu)] was detected in the euthanized fish (Fig. 1A), suggesting that the signs of discomfort were due to the progression of the *M. marinum* infection rather than the immunosuppression itself. In line with this, the feeding of dexamethasone did not cause signs of discomfort in uninfected fish. Therefore, the increase in the bacterial counts following a dexamethasone treatment is likely to be caused by the progression of a latent mycobacterial infection into an active phase.

The kinetics of this progression were analyzed by measuring the bacterial numbers at several time points following a dexamethasone treatment in two independent experiments (Fig. 1B; Fig. S1A). An increase in the bacterial burden was observed 3 days after the onset of the feeding of dexamethasone (*P*=0.007, one-tailed Mann–Whitney test), eventually leading to an approximately 1000-fold increment at 4 weeks. A higher dose of dexamethasone (20 µg/day/fish) had an essentially identical effect on bacterial numbers (Fig. S1B). Therefore, the lower dose of dexamethasone (10 µg/day) was selected for subsequent experiments. A pretreatment with dexamethasone prior to infection did not affect the outcome of a low-dose infection (Fig. S1C), suggesting that dexamethasone is more likely to affect the host's ability to control a latent infection than to suppress the responses against mycobacteria during an early infection. To obtain a more mechanistic insight into this, we next investigated the cellular and molecular events associated with the reactivation of a latent mycobacterial infection in the adult zebrafish.

### Reactivation of a latent mycobacterial infection alters the quality and quantity of granulomas

**Fig. 2.**
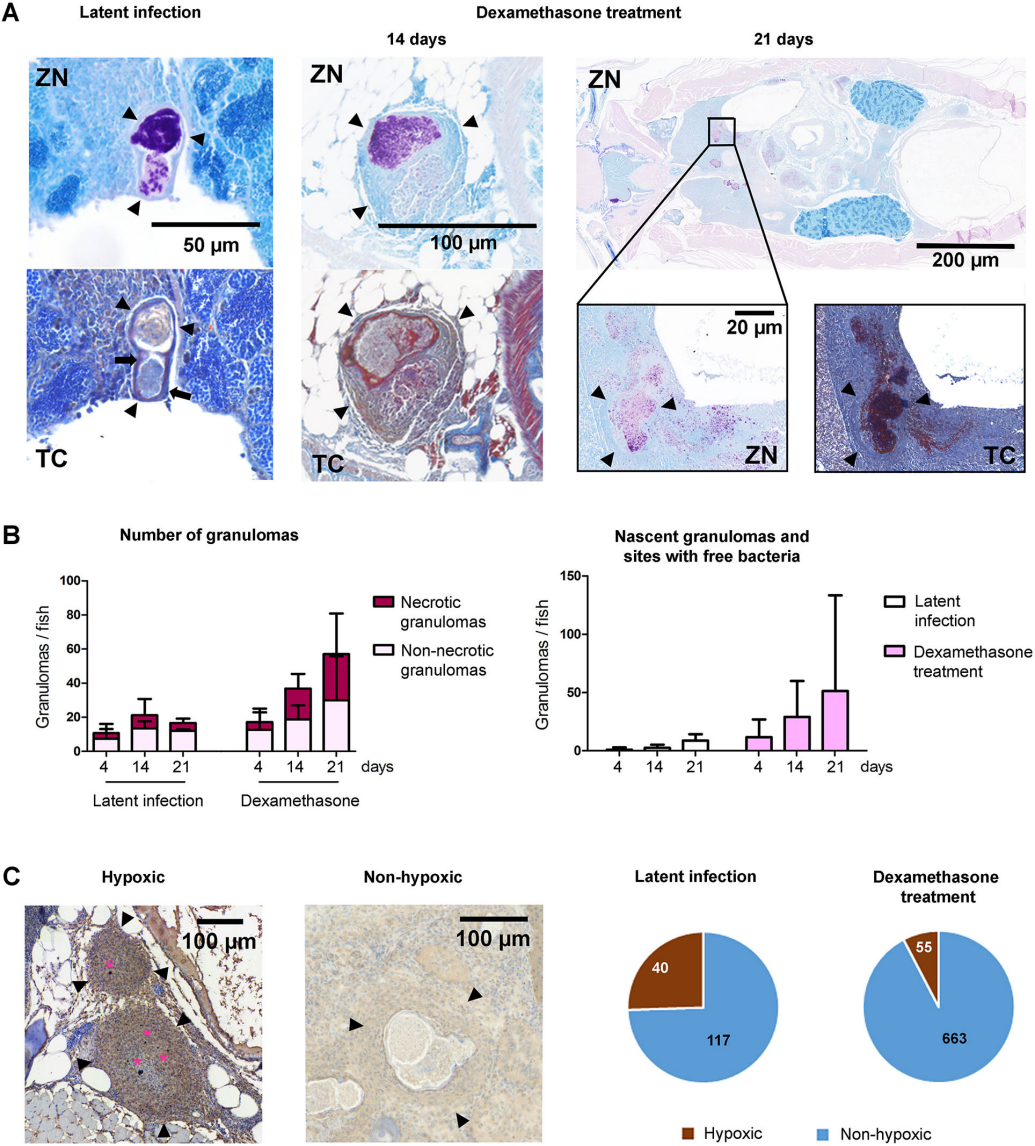
**Dexamethasone treatment leads to disruption of the granuloma structure, increase in the number of granulomas and loss of hypoxia in zebrafish with a latent mycobacterial infection.** (A) Ziehl–Neelsen staining (ZN) of mycobacteria (purple). Black arrowheads indicate the outline of a granuloma. Trichrome staining (TC) of fibrous tissue around granulomas (blue) (black arrows). (B) Quantification of the total number of granulomas and the proportion of necrotic and non-necrotic granulomas (left), and the number of nascent granulomas and sites with non-capsulated mycobacteria (right) per fish after 4, 14 and 21 days of dexamethasone treatment or normal feeding (*n*=3-6 fish/group). Data are presented as mean±s.d. Statistical significance was assessed by unpaired Student's *t*-test. (C) Hypoxic staining shows hypoxic lesions inside granulomas in dark brown (pink stars). The proportions of hypoxic and non-hypoxic granulomas during a latent infection, and after 2 weeks of dexamethasone treatment, are quantified in the pie chart. See also Fig. S2.

The formation of granulomas is defined as the hallmark of tuberculosis, and granulomas also form in the adult zebrafish during the course of an *M. marinum* infection. To demonstrate the presence of granulomas during reactivation, we used Ziehl–Neelsen staining to visualize mycobacteria in histological sections, and trichrome staining to visualize the fibrous capsule typically found around mature granulomas (Parikka et al., 2012). In fish with a latent infection, the granulomas were generally well organized and surrounded by a thick layer of fibrous tissue confining the mycobacteria inside the granulomas (Fig. 2A). After 2 weeks of dexamethasone treatment, the granulomas appeared larger and looser in structure, showing thinning and disruption of the surrounding fibrous layer. After 3 weeks of dexamethasone treatment, further loss of granuloma integrity was observed, together with bacteria escaping from the granulomas and spreading into tissues, indicating progression of the infection into an active state.

To quantify the dexamethasone-induced changes in the number and types of granulomas, we counted the total number of granulomas per fish at different time points during the treatment (4, 14 and 21 days, *n*=3-6) (Fig. 2B). In the control group, the number of granulomas remained relatively constant (average of 9-17 granulomas/fish), while in the dexamethasone-treated group, the number of granulomas increased from an average of 17 granulomas at day 4 to an average of 57 granulomas per fish at 21 days. To further characterize the process, we calculated the number of necrotic (Fig. 2B) and multicentric granulomas separately (Fig. S2A-C). Both are subtypes of mature granulomas that contain a high number of mycobacteria, indicating an advanced stage of a mycobacterial disease (Parikka et al., 2012). We found that after 3 weeks of dexamethasone treatment, the number of necrotic granulomas had increased from an average of 4.5 at day 4 to 27 at day 21 (*P*=0.02, one-tailed Mann–Whitney test) (Fig. 2B), while there was also an increase in the number of multicentric granulomas (Fig. S2B) and the proportion of necrotic versus non-necrotic granulomas (Fig. S2C). Consistent with the qualitative analysis, there was also a clear increase in the number of nascent granulomas and sites with noncapsulated mycobacteria, from a mean of 11 granulomas or sites at day 4 to 51 at day 21 (Fig. 2B). This suggests that the treatment leads to the escape of mycobacteria from existing granulomas and the seeding of new ones.

Hypoxia has been shown to have an important role in mycobacterial pathogenesis. A low-oxygen environment induces metabolic adaptation in bacteria, which allows the bacteria to persist inside the host during a latent infection. To visualize hypoxic areas in granulomas, pimonidazole treatment and Hypoxyprobe staining were used (Fig. 2C) (Matty et al., 2015). Small hypoxic areas were found adjacent to 25% of the granulomas in the fish with a latent infection. Following a 21-day dexamethasone treatment, hypoxic lesions were observed in only 8% of all granulomas (*P*<0.0001, Fisher's exact test) (Fig. 2C).

Altogether, these data suggest that during the reactivation of a latent disease, mycobacteria first replicate within existing granulomas. This leads to bacteria escaping and forming new granulomas, as well as the appearance of free bacteria, which is associated with the disruption of the granuloma structure and the loss of hypoxia inside the granulomas.

### Dexamethasone treatment leads to a decrease in the amount of lymphocytes

**Fig. 3.**
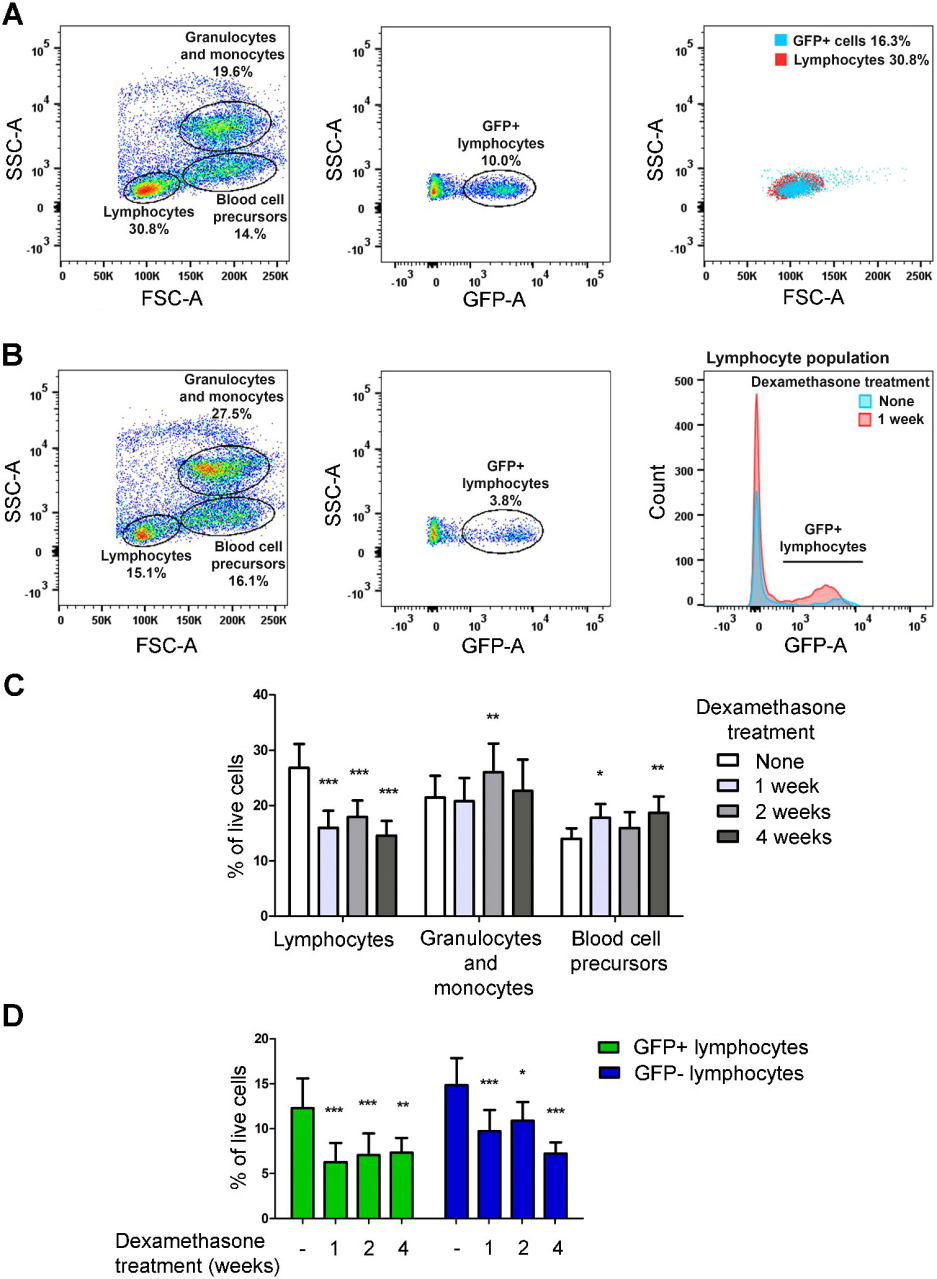
**Dexamethasone treatment leads to a depletion of lymphocytes in the adult zebrafish.** (A) Sorting of the kidney cell populations of *lck-GFP* fish based on size (side scatter, SSC-A) and granularity (forward scatter, FSC-A) (left). The lymphocyte population is further separated into GFP^+^ and GFP^–^ subpopulations (middle), and the majority of the GFP^–^ cells fall into the lymphocyte gate by the SSC-A and FSC-A. (B) After a 1-week dexamethasone treatment, the kidney cells fall into the same gates, but the number of both GFP^+^ and GFP^–^ lymphocytes is depleted (right), as quantified in C and D. *n*=12. Data are presented as mean±s.d. Statistical significance is analyzed by two-way ANOVA with Bonferroni posttest, *P<0.05, **P<0.01, ***P<0.001. Also see Figs S3 and S4.

Next, we investigated the alterations caused by dexamethasone in immune cell populations in the kidney, which is the site of hematopoiesis in the adult zebrafish. For this, we first treated wild-type fish with dexamethasone for 1, 2 and 6 weeks and dissected their kidneys for a flow cytometric [fluorescence-activated cell sorting (FACS)] analysis. For the analysis, the cells in the live gate were separated into populations based on size [defined by the forward scatter (FSC)] and granularity [side scatter (SSC)]. These included lymphocytes, granulocytes and monocytes, and blood cell precursors. Upon dexamethasone treatment, the proportion of lymphocytes decreased from 19.3±3.5% to 12.4±1.6% (*P*<0.01, two-way ANOVA), while the relative amount of granulocytes and monocytes and blood cell precursors remained unchanged (Fig. S3). To study the effect of dexamethasone treatment on lymphocytes in more detail, we used the Tg:lck(*lck-EGFP*) fish line, which expresses *GFP* under the tyrosine kinase promoter specific for mature T cells, NK-like cells and myeloid-like cells in the kidney of adult zebrafish (Langenau et al., 2004; Carmona et al., 2017). *lck-GFP* fish were treated with dexamethasone for 1, 2 and 4 weeks, followed by FACS analysis of the kidney cell populations as above (Fig. 3; Fig. S4B). As expected, the GFP^+^ cell population mainly fell into the lymphocyte gate (Fig. 3A; Fig. S4B,C) (Langenau et al., 2004). Again, upon dexamethasone treatment, the proportion of lymphocytes decreased from 30.1±4.0% to 18.2±3.1% (*P*<0.001, two-way ANOVA), while there was a slightly increasing trend in the relative amount of granulocytes and monocytes and blood cell precursors (Fig. 3B,C; Fig. S4B,C). More specifically, the proportion of GFP^+^ lymphocytes decreased during the first week of dexamethasone treatment, from the original 12.5±3.4% of live cells in untreated fish to 6.3±2.4% (*P*<0.001, two-way ANOVA), after which it remained the same throughout the treatment (Fig. 3D). In addition, the GFP^–^ lymphocyte population, which contains B cells and immature T cells (Langenau et al., 2004), decreased from 18.5±2.8% to 10.3±1.7% of live cells (*P*<0.001, two-way ANOVA) after 4 weeks of treatment (Fig. 3D). The proportion of GFP^+^ lymphocytes compared to GFP^–^ ones decreased slightly at the 1- and 2-week time points, but by 4 weeks, the GFP^+^: GFP^–^ ratio had returned to the same as that in the control group (40.2% and 43.0% of GFP^+^ lymphocytes) (Fig. S4D).

### Dexamethasone treatment alters the expression of T cell markers upon infection

**Fig. 4.**
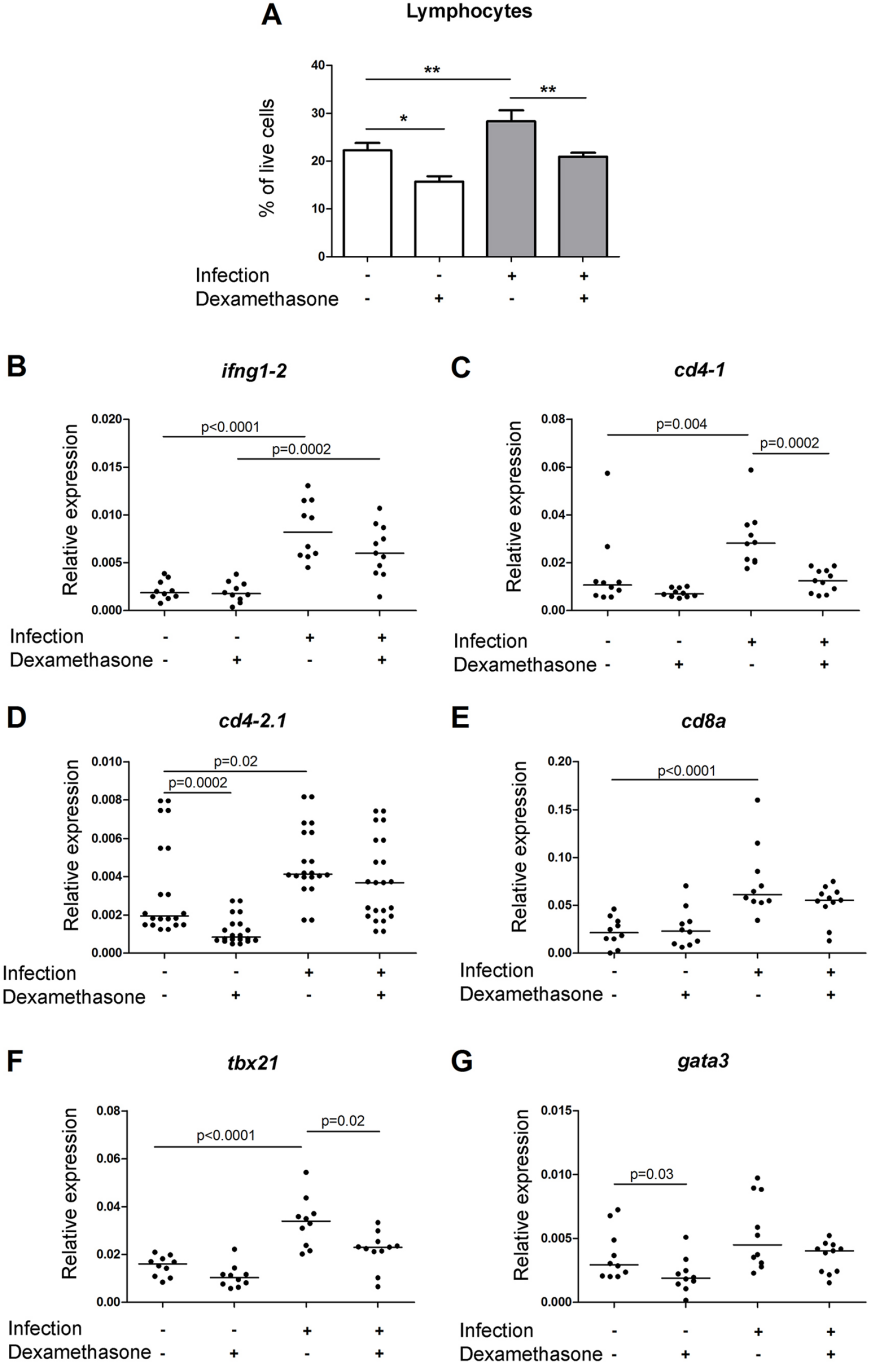
**Dexamethasone treatment decreases the expression of CD4^+^ T lymphocyte markers in uninfected and infected zebrafish.** (A) Lymphocytes are expanded in the zebrafish kidney upon a latent *M. marinum* infection and depleted by dexamethasone treatment. The kidney cell populations of AB fish were analyzed with FACS. Bars show the mean percentage of lymphocytes±s.d. *P*-values were calculated using two-way ANOVA with Bonferroni posttest, *P<0.05, **P<0.01, ***P<0.001. (B-G) Relative expression levels of inflammatory genes and T cell markers. Each dot represents the expression level of the marker gene relative to the housekeeping gene (*EF1a*). Horizontal lines show the median value in each group. *n*=10-11 fish/group. *P*-values were calculated using the two-tailed Mann–Whitney test. (B) *ifng1-2*, (C) *cd4-1*, (D) *cd4-2.1*, (E) *cd8a*, (F) *tbx21*, (G) *gata3*. See also Fig. S5.

To further characterize the effects of dexamethasone treatment on the immune response against mycobacteria, we compared the kidney lymphocyte population of uninfected and infected wild-type zebrafish after 1 week of treatment with dexamethasone (Fig. 4; Fig. S5). A latent mycobacterial infection led to an increase in the lymphocyte population (from 21.7±4.5% to 28.3±7.3%; *P*<0.01, two-way ANOVA with Bonferroni posttest). Treating fish with a latent infection with dexamethasone led to a decrease in the proportion of lymphocytes, to the level seen in uninfected fish (24.6±3.2%, *P*<0.05, two-way ANOVA with Bonferroni posttest) (Fig. 4A).

To investigate the effect of dexamethasone on lymphocyte function, we studied the expression levels of marker genes of different types of T and B cells from the isolated kidney cells using quantitative reverse transcription PCR (qRT-PCR) (Fig. 4B-G; Fig. S5B-G). Of the studied inflammation markers, both *tnfa* and *interferon gamma 1-2* (*ifng1-2*; *ifng-1*) were induced in the fish carrying a latent infection, with the former being slightly upregulated (Fig. S5B), and the latter slightly downregulated (Fig. 4B), by dexamethasone. The expression of *tnfa* correlated with the bacterial burden (Spearman r 0.8180, *P*<0.0001), which likely explains its relative elevation in the dexamethasone-treated individuals, whereas no such correlation was observed for *ifng1-2* expression (r –0.1740).

Zebrafish have CD4^+^ and CD8^+^ lymphocyte populations with functions similar to those in humans (Langenau et al., 2004; Yoon et al., 2015; Renshaw and Trede, 2012). Consistently, the expression of both *cd4-1* and *cd4-2.1* was induced upon infection (*P*=0.004 and *P*=0.0002, respectively), whereas their expression was reduced following dexamethasone treatment: *cd4-1* was suppressed following reactivation (*P*=0.0002) (Fig. 4C), and *cd4-2.1* was reduced in both uninfected (*P*=0.02) and infected (*P*=0.088) fish (Fig. 4D). Expression of *cd8a*, a marker gene for cytotoxic T cells, was induced in infected fish (*P*<0.0001), but its expression was not altered by the dexamethasone treatment (Fig. 4E).

The balance between the CD4^+^ Th1 and Th2 cells has been shown to play a role in the control of a mycobacterial infection (Hammarén et al., 2014). To study the CD4^+^ subpopulation more specifically, we used the expression of the *tbx21* transcription factor as a marker for the Th1 population, and the transcription factor *gata3* and *il4*, *il10* and *il13* as markers for Th2 cells. *tbx21* expression was induced upon infection (*P*<0.0001) (Fig. 4F), as was the expression of *il10* (*P*=0.004) (Fig. S5C), while the expression of the other Th2 markers tested remained relatively constant (Fig. S5D,E). Dexamethasone treatment suppressed the expression of *tbx21* in uninfected fish (*P*=0.052) and in fish with a latent infection (*P*=0.02) (Fig. 4F). Of the Th2 markers, a decreasing expression for *gata3* was seen after dexamethasone treatment in uninfected fish (*P*=0.03) and a corresponding trend was observed in infected fish (Fig. 4G). The expression of *IgM* (*ighm*), which was used as a marker for B cells, remained relatively constant in both infected and dexamethasone-treated fish (Fig. S5F), whereas *tgfb1b*, a marker for regulatory T cells (Tregs), was suppressed upon infection in the immunocompetent fish (*P*=0.0007), and upregulated in the dexamethasone-treated group (*P*=0.03) (Fig. S5G). However, the expression of *foxp3a*, a transcription factor characteristic for Tregs was not affected by dexamethasone treatment (Fig. S4H).

Overall, these data indicate that a dexamethasone treatment leads to the general depletion of lymphocytes. The most prominent effect is seen in the expression of *cd4-1*, *cd4-2.1* and the Th1 marker *tbx21*. Although dexamethasone-treated fish are able to induce an IFN-γ response against mycobacteria, the suppression of specific T cell populations, together with the induction of the inhibitory cytokine *tgfb1b*, are associated with compromised ability to control infection.

### The zebrafish model can be utilized to assess the efficacy of drugs at different phases of a mycobacterial infection

Owing to their small size and fast reproduction rate, zebrafish are well suited for large-scale pharmaceutical screening studies (Lohi et al., 2013). On this premise, we investigated whether the dexamethasone-based reactivation model can be used for testing the effectiveness of antimicrobial medicines against reactivated mycobacteria.

**Fig. 5.**
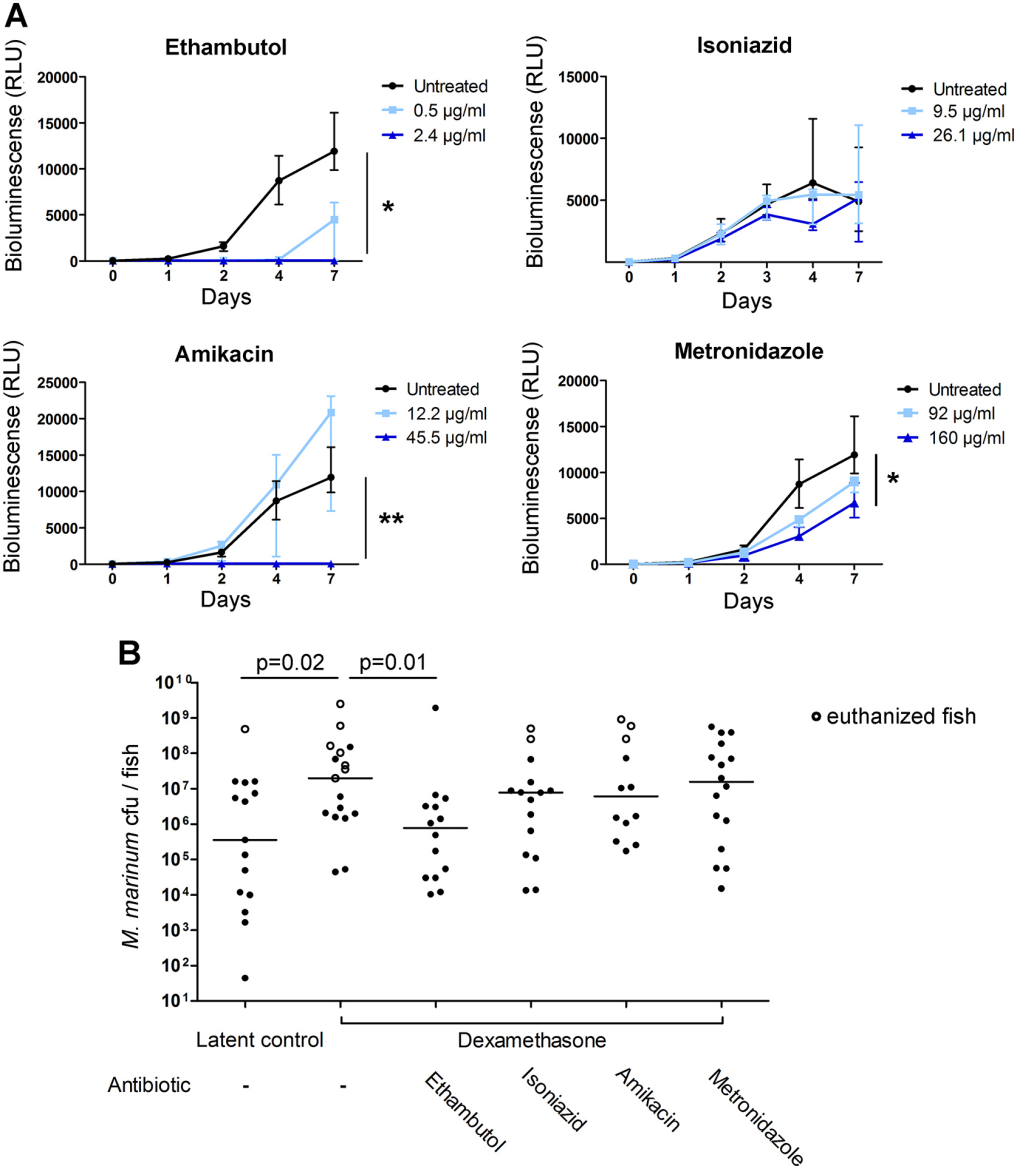
**Ethambutol inhibits the growth of *M. marinum* both *in vitro* and upon dexamethasone treatment *in vivo*.** (A) The effects of selected antibiotics on the growth of a bioluminescent strain of *M. marinum in vitro*. The graphs represent the detected relative bioluminescence (RLU) of bacteria at the indicated time points after exposing them to an antibiotic. *n*=5-6. One-way ANOVA with Friedman's test and Dunn's multiple comparison test was used for statistical analysis, *P<0.05, **P<0.01. (B) Adult zebrafish with a latent mycobacterial infection were treated for 3 weeks with dexamethasone (10 µg/fish/day) followed by 4 weeks of treatment with selected antibiotics. Each dot represents the bacterial burden per fish. Horizontal lines show the median value of each group. The statistical analysis was performed with the two-tailed Mann–Whitney test.

We chose four different antimicrobial drugs that have been used for treating mycobacterial diseases, including ethambutol, isoniazid, amikacin and metronidazole. Each of the antibiotics was first tested for their effect against *M. marinum in vitro* using a bioluminescent strain, which allowed the quantification of bacterial growth at different time points with a luminometer. Two different doses were tested for each antimicrobial drug. During the 7-day follow-up period, all antibiotics, excluding isoniazid, inhibited the growth of *M. marinum* in a dose-dependent manner, compared to bacteria cultured in medium without antibiotics (Fig. 5A). Higher doses of ethambutol (2.4 µg/ml), and amikacin (45.5 µg/ml), completely prevented bacterial growth (*P*<0.05, respectively, one-way ANOVA with Friedman's test).

Next, zebrafish with a latent *M. marinum* infection were treated for 3 weeks with dexamethasone, followed by 4 weeks of treatment with antibiotics (50 µg/fish/day each). Metronidazole (160 µg/ml), which is designed to target anaerobic microbes, inhibited the growth of *M. marinum in vitro* by 48% compared to controls, but did not limit the bacterial burden *in vivo* in a latent (Fig. S6A) or reactivated infection (Fig. 5B).

However, ethambutol decreased the median bacterial counts in dexamethasone-treated zebrafish by 96% compared to untreated controls (*P*=0.01, Mann–Whitney test), which is close to the level seen in a latent infection (Fig. 5B). Of note, a 2-week treatment with ethambutol did not affect bacterial counts in zebrafish with a latent *M. marinum* infection (Fig. S6B), consistent with the knowledge that ethambutol acts by preventing the replication of mycobacteria (Forbes et al., 1962). This further demonstrates that dexamethasone treatment causes the reactivation of a latent mycobacterial infection in zebrafish, leading to the active replication of the bacteria and susceptibility to ethambutol.

### Identification of novel protective postexposure vaccine antigens against the reactivation of TB in the zebrafish model

We have previously shown that the zebrafish-*M. marinum* infection model is suitable for the preclinical screening of novel vaccine candidates against a mycobacterial infection (Oksanen et al., 2013, 2016). The zebrafish can be partially protected against a primary *M. marinum* infection by BCG, or by prophylactic DNA-based vaccines consisting of a combination of previously studied mycobacterial antigens (Ag85 and ESAT-6), as well as with some novel antigens (Myllymäki et al., 2017).

**Fig. 6.**
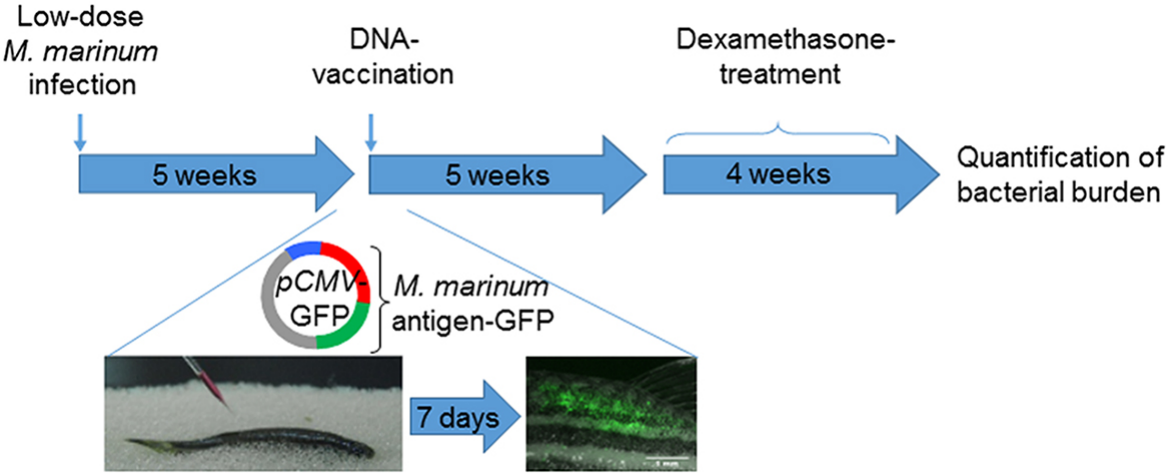
**Schematic representation of the experimental protocol for testing vaccine candidates against the reactivation of a latent mycobacterial infection.** Fish with a latent *M. marinum* infection are immunized with a DNA plasmid carrying a GFP-tagged mycobacterial antigen, which allows detection of successful immunizations by fluorescent microscopy. After 5 weeks, the fish are subjected to dexamethasone treatment (10 μg/fish/day) for 4 weeks, followed by determination of bacterial burdens by qPCR.

**Fig. 7.**
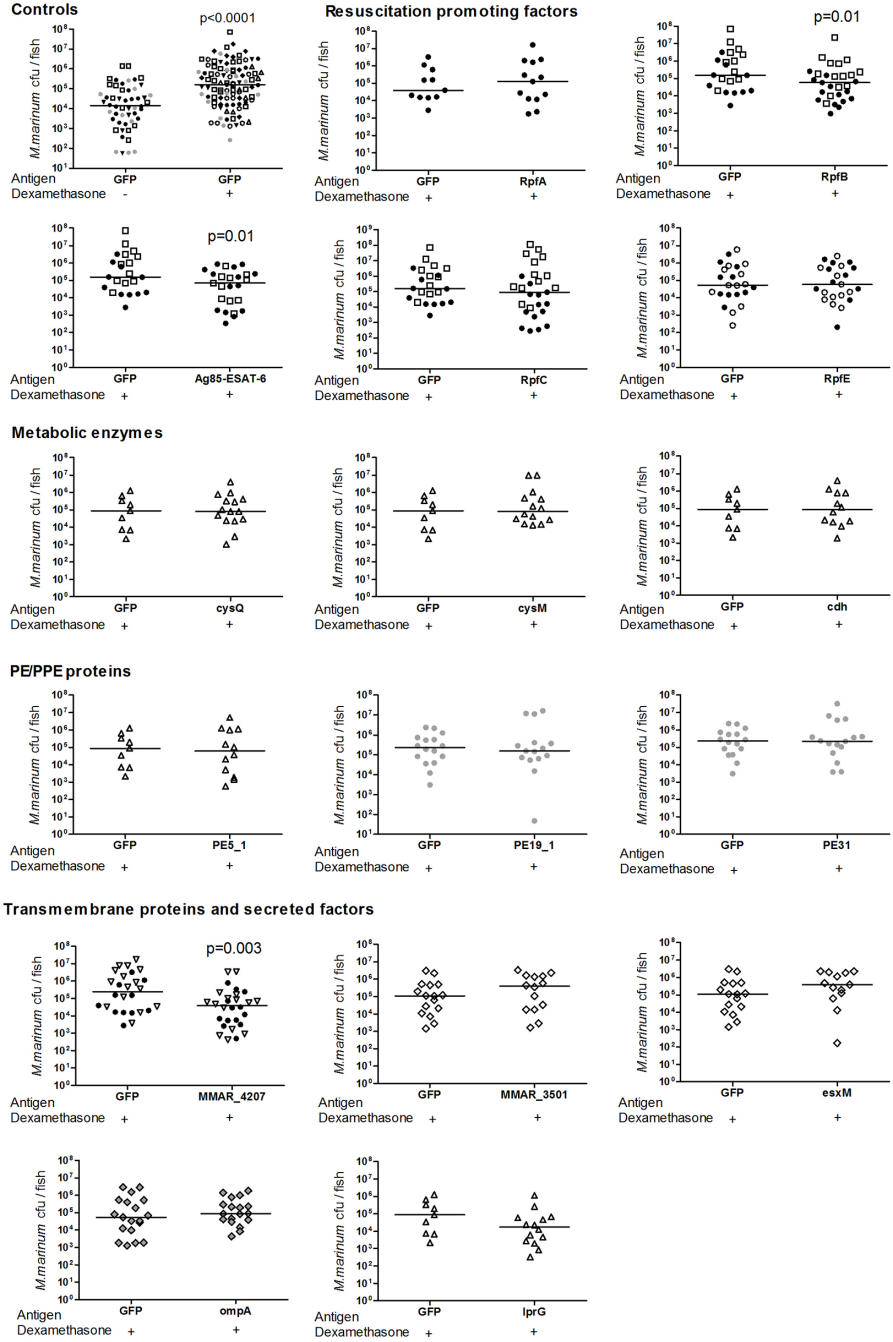
**Ag85-ESAT-6, RpfB and MMAR_4207 antigens decrease bacterial burdens upon reactivation of a latent *M. marinum* infection in the adult zebrafish.** Fish with a latent *M. marinum* infection were immunized with the GFP control or experimental vaccines and 5 weeks later treated for 4 weeks with dexamethasone. Each dot represents the bacterial load in one fish. Horizontal lines show the median values of pooled experiments (*n*=11-29 fish/group). Results from different experiments are marked with different symbols. A two-tailed Mann–Whitney test was used to calculate *P*-values.

Based on this, we studied the applicability of the dexamethasone-based reactivation model for the preclinical screening of new DNA-based vaccines by examining the therapeutic effect of the selected mycobacterial antigens against the reactivation of a latent *M. marinum* infection. The zebrafish were first infected with a low dose of *M. marinum*, and vaccinated 5 weeks postinfection. Four weeks after the vaccinations, the fish were subjected to dexamethasone treatment (10 µg/fish/day) for 4 weeks, after which their bacterial burdens were quantified by qPCR (the protocol is outlined in Fig. 6). The antigens showing a significant reduction in bacterial counts in the initial screen were subjected to a replicate experiment to confirm the effect. We first tested the effect of the Ag85-ESAT-6 antigen combination against reactivation, and found that this immunization decreased the bacterial counts by 56% or 0.35 log_10_ (*P*=0.02, two-tailed Mann–Whitney test) compared to the control group immunized with an empty *GFP* plasmid (Fig. 7).

The other antigens tested involved genes from different functional categories, including the four *M. marinum* Resuscitation promoting factors (Rpf proteins), three members of the PE/PPE protein family, four conserved membrane proteins, one secreted factor and three metabolic enzymes. Compared to the GFP-immunized control group, most of these did not affect the bacterial burden of the fish upon reactivation. Noteworthy, immunization with the antigens consisting of RpfB and MMAR_4207, led to a significant reduction in bacterial counts (by 63% or 0.53 log_10_ and 85% or 0.81 log_10_, respectively) (Fig. 7).

Altogether, these data show that the dexamethasone-based reactivation model provides an amenable tool for the preclinical screening of both novel antimicrobials and therapeutic vaccines against the reactivation of TB. For the latter, we identified two promising candidates, RpfB and the conserved membrane protein MMAR_4207.

## DISCUSSION

TB remains a major health problem, and new antimicrobial medicines and vaccines are needed to prevent new infections and to prevent the reactivation of a latent TB infection (WHO, 2017). During the past decade, the zebrafish, with its natural pathogen *M. marinum*, has become a powerful model for studying the mechanisms associated with mycobacterial defense as well as new therapeutic strategies to fight mycobacterial infections. Some of the results have been already translated into combatting human TB infections (Adams et al., 2014; Tobin et al., 2013, 2012; Oehlers et al., 2017). In the current study, we utilized the adult zebrafish-*M. marinum* model to establish a tool for studying the control and reactivation of latent mycobacterial infections. This was achieved by the administration of the glucocorticoid dexamethasone in the fish food. In humans, glucocorticoids are used as immunosuppressants to treat various diseases, such as arthritis, and long-term use at a high dose has been associated with the reactivation of a latent TB infection, but the exact mechanisms underlying the reactivation are poorly understood (Jick et al., 2006).

TB remains one of the major killers of HIV-coinfected individuals: people with HIV are 20-30 times more likely to develop active TB; and there were 0.4 million of such deaths in 2015 (WHO, 2017). In humans and macaques, an HIV or a Simian immunodeficiency virus (SIV) infection is associated with the loss of CD4^+^ T cells, which has long been thought to be the main factor promoting the reactivation of a latent TB infection (Barnes et al., 1991; Diedrich et al., 2010). This is also supported by data from the mouse model (Kupz et al., 2016a,b). Using the adult zebrafish, we observed a depletion of the lymphocyte population by dexamethasone treatment in uninfected fish, and a failure to induce lymphocyte expansion in infected fish. This is consistent with the depletion of T cells by dexamethasone seen in zebrafish larvae (Langenau et al., 2004). Moreover, our results suggest that dexamethasone, in particular, decreases the amount of conventional CD4^+^ lymphocytes, including both Th1 and Th2 cells, whereas the expression of *tgf-b1b*, induced by the immunosuppressive Treg cell population, is upregulated. Dexamethasone has been shown to alleviate allergic symptoms in mice by upregulation of Treg cells (Zhang et al., 2016), but the exact role of these cells and the associated cytokines in the control of mycobacterial infections remains to be studied.

Considering the functional similarities between human and zebrafish CD4^+^ and CD8^+^ T cells (reviewed in Renshaw and Trede, 2012), CD4^+^ cells could be a key player in controlling a latent mycobacterial infection also in the zebrafish. However, the susceptibility to the reactivation of a latent TB varies even among HIV-coinfected individuals, and in a macaque model, CD8^+^ and B cells have been shown to elicit a protective effect in some of the coinfected individuals (Foreman et al., 2016). In the zebrafish, the expression levels of *cd8a* and the B cell marker *IgM* remained constant during the dexamethasone treatment, suggesting that these cells are left unaffected by the treatment. Furthermore, the individual zebrafish within a single line have been shown to differ in their ability to control a mycobacterial infection, probably due to genetic heterogeneity, leading to differences in the immune response (Hammarén et al., 2014). We also observed that some fish managed to maintain a relatively low mycobacterial count despite the dexamethasone treatment, which could be explained by the protective effect of CD8 and/or B cells, mimicking the situation in humans and macaques (Foreman et al., 2016; van Meijgaarden et al., 2015). The number of granulocytes and monocytes was not decreased by dexamethasone. Macrophages and neutrophils are needed for the formation of granulomas and for the early control of a mycobacterial infection (Davis et al., 2002; Yang et al., 2012; Davis and Ramakrishnan, 2009). Although treatment with dexamethasone prior to an infection does not increase susceptibility to mycobacteriosis, but rather compromises the control of a chronic infection, confirming that dexamethasone does not affect the properties of macrophages or neutrophils would require functional assays. In the infected, dexamethasone-treated fish, we observed a strong negative correlation (Spearman r –0.88, *P*=0.0007) between the bacterial burden and the relative amount of macrophages and neutrophils in the kidney, but it remains elusive whether there is any causal relationship in either direction.

In adult zebrafish, granuloma structures reflect the stage of the disease (Parikka et al., 2012), and, similar to observations in macaques, rabbits and guinea pig, hypoxia is observed in granulomas in adult zebrafish with a latent *M. marinum* infection, mainly in the cells adjacent to necrotic areas (Via et al., 2008; Oehlers et al., 2015). Upon reactivation, we observed loss of hypoxia, which was associated with increasing granuloma counts, their necrosis and multicentricity, and disruption of the surrounding fibrous capsule, together with the emergence of disseminated bacteria at several sites within tissues and the formation of nascent granulomas. The latter are normally associated with a recent infection that has not yet reached a latent stage (Parikka et al., 2012), but, in the context of dexamethasone treatment, their appearance likely indicates progression of the infection from a latent state into an active one. As the mechanisms that trigger the exit of mycobacteria from the granuloma remain largely elusive (Boggiano et al., 2017), this model provides a feasible tool for examining the role of different immune cells in the control of mycobacterial infections.

Owing to their cell wall properties and ability to persist inside granuloma structures, mycobacteria have been a difficult pathogen to target by current antibiotics (Peddireddy et al., 2017). Multidrug-resistant strains present a further challenge for treatments, and new antimicrobial drugs are therefore urgently needed (WHO, 2017). We observed that ethambutol effectively prevented the growth of *M. marinum in vitro*, and after dexamethasone treatment, mycobacteria could be successfully targeted *in vivo* with ethambutol, whereas a 2-week treatment had no effect on a latent infection. Of the other antibiotics tested in this study, a larger dose of amikacin could inhibit mycobacterial growth *in vitro*, thus a larger dose could improve the result *in vivo* as well. Metronidazole is designed to exhibit its antibacterial activity under anaerobic conditions. It has been shown to prevent the reactivation of latent TB in the macaque model (Lin et al., 2012), but not in the guinea pig (Hoff et al., 2008), and the human trial was discontinued owing to neurotoxicity (Carroll et al., 2013). In our hands, metronidazole inhibited the growth of *M. marinum in vitro* by ∼50%, but the tested dose was ineffective against both a latent and a reactivated infection in zebrafish. This could be due to a lack of anaerobiosis (despite the presence of hypoxic lesions) in the zebrafish granulomas.

In previous studies, we showed that the zebrafish-*M. marinum* infection model is feasible for use in the preclinical screening of vaccines against a mycobacterial infection (Oksanen et al., 2013; Myllymäki et al., 2017). This was also assessed in the current study in the context of preventing reactivation of a latent mycobacterial disease. We screened the effectiveness of an antigen combination Ag85B-ESAT-6, which has been studied in HIV-positive Mtb carriers (Reither et al., 2014), together with 15 novel conserved mycobacterial antigens. We found that the Ag85B-ESAT-6 combination was able to improve the control of dexamethasone-activated infection, reducing the median bacterial burden by 85%. Importantly, two of the tested novel mycobacterial antigens, namely RpfB and MMAR_4207, significantly decreased the bacterial burden in the dexamethasone-treated fish. Their protective effect is comparable to the effect of the BCG vaccination in a primary *M. marinum* infection in the zebrafish (82% or 0.76 log_10_ reduction in bacterial burden) (Oksanen et al., 2016), or an *M. tuberculosis* infection in mice (0.35-1.2 log_10_ reduction in bacterial counts) (Jeon et al., 2008). As its name implies, RpfB is thought to promote the resuscitation and growth of dormant, nongrowing cells, and the potential of the Rpf proteins as vaccine antigens has been studied to some extent (Romano et al., 2012); thus, the promising result is not completely unexpected. MMAR_4207 is a conserved membrane protein with an unknown function, which makes this antigen an interesting novel candidate for further studies. In our previous study, these antigens did not affect the bacterial burden as prophylactic vaccines in a primary infection assay (Myllymäki et al., 2017), suggesting that they would more effectively target mycobacteria upon reactivation. However, it is also possible that these vaccines reduce the bacterial load during the latent phase of infection before the immunosuppression takes place. Therefore, determining the exact mechanism of their action requires further studies. Vaccine candidates that elicit a strong CD4-based response generally provide a rather low level of protection (Tameris et al., 2013; Sakai et al., 2016), and, recently, other protection mechanisms, such as the CD8^+^ and B cells and the humoral responses, have also received attention (Achkar et al., 2015; van Meijgaarden et al., 2015; Kamath et al., 2006). A model system, where the CD4 arm of immunity is hampered, could provide an interesting means to explore these. Moreover, a therapeutic vaccine that would improve the control of a latent TB, also in the immunocompromised, would be a great asset in the battle against TB. Based on the results of this study, the zebrafish model can provide a time- and cost-effective tool for the early screening of such vaccine candidates, followed by studies in larger animal models, such as macaque SIV-TB coinfection (Diedrich et al., 2010; Foreman et al., 2016; Capuano et al., 2003).

In conclusion, the results of this study show that a latent *M. marinum* infection can be reactivated with dexamethasone treatment in the adult zebrafish. We used this method to characterize the mechanisms associated with the process and found that in zebrafish, like in humans, the depletion of lymphocytes, especially the CD4^+^ T cell population, is associated with the impaired control of a latent mycobacterial infection. This method also provides a tool for screening the effectiveness of novel antimicrobial drugs and vaccines against the reactivation of a latent mycobacterial infection. Importantly, we identified two novel mycobacterial antigens – RpfB and MMAR_4207 – that show protection against the reactivation of a latent mycobacterial infection as postexposure DNA vaccines.

## MATERIALS AND METHODS

### Zebrafish

In the experiments, 5- to 9-month-old, wild-type AB zebrafish (*Danio rerio*) from the Tampere Zebrafish Core Facility were used. In addition, for the flow cytometry, adult Tg:lck(*lck-EGFP*) fish [obtained from the Zebrafish International Resource Center (ZIRC), University of Oregon, USA] were used. Fish were maintained in a 10 h/14 h light/dark cycle, in a flow-through salt water system at 28°C and were fed twice daily. The Animal Experiment Board in Finland approved all animal experiments (ESAVI/8125/04.10.07/2013) and studies were carried out in accordance with the EU directive 2010/63/EU on the protection of animals used for scientific purposes. Humane endpoint criteria were applied throughout all experiments, the wellbeing of the fish was monitored carefully, and any fish showing signs of illness or discomfort were immediately euthanized with 0.04% 3-aminobenzoic acid ethyl ester pH 7.0 (A5040, Sigma-Aldrich, St Louis, MO, USA).

### Experimental infections

The *M. marinum* strain ATCC 927 was used for the infections. The culturing of bacteria and infections were carried out as described earlier (Parikka et al., 2012), except that bacteria were diluted with sterile 0.9× phosphate buffered saline (PBS) and 0.03 mg/ml Phenol Red before the infections. The infection dose was 33±21 cfu/fish in all experiments, and was confirmed by plating on 7H10 Middlebrook OACD agar plates (BD Biosciences, Franklin Lakes, NJ, USA).

### Preparation and administration of medicine food

Immunosuppressive agents and antibiotics were administrated per os in the fish food. The gelatin-coated fish food contained 2.5 mg/g of the immunosuppressant or 12.5 mg/g of the antibiotics. Bovine gelatin (G9391, Sigma-Aldrich; 400 mg) was diluted in 5 ml distilled water by heating (45°C). Then, 25 mg of each immunosuppressant (azathioprine A4638, dexamethasone D4902, prednisolone P6004 and methylprednisolone M1755200, Sigma-Aldrich) or 125 mg of antibiotic (ethambutol E4630, metronidazole M3761, isoniazid I3377 and amikacin A0368000, Sigma-Aldrich) was mixed with 2 ml of 70% EtOH and the suspension was combined with the gelatin solution. The gelatin mixture was spread evenly on 10 g of dry fish food pellets (SDS400, Special Diet Services, Essex, UK) and air dried overnight. The food was weighed and homogenized using a mortar and a pestle. The increase in the weight of the food, from the added gelatin and medicines, was taken in account when the daily doses of prepared food were calculated and weighed, so that each fish received 4 mg of food containing the desired dose of the drug. During the reactivation treatments, the fish were fed once daily. The water circulation was stopped during the feeding to avoid the spreading of chemical-containing food into other tanks of the unit.

### Histology

For histological staining, fish with a latent infection were treated with dexamethasone (10 µg/fish/day) and samples were collected after 4, 14 and 21 days of treatment (*n*=3-6 fish/group). Untreated groups of fish were collected as controls at each time point. The fish were euthanized with 0.04% 3-aminobenzoic acid ethyl ester (A5040, Sigma-Aldrich) and fixed in 10% phosphate buffered formalin (Oy FF-Chemicals Ab, Haukipudas, Finland) for 7 days. Decalcification, embedding into paraffin and the cutting of sections were performed according to Parikka et al. (2012). Two parallel 5-µm sections were collected every 200 µm on two SuperFrost^®^Plus glasses (Thermo Fisher Scientific), deparaffinized and used for either Ziehl–Neelsen or trichrome staining. Ziehl–Neelsen staining was performed as described in Parikka et al. (2012). The trichrome staining protocol was a modified version from Mallory's trichrome staining; before treatment with acid fuchsin, the sections were incubated in Bouin's solution (HT10132, Sigma-Aldrich) overnight at room temperature. All slides were scanned with an Olympus BX43 microscope and analyzed with the JPEG2000 virtual slide and ImageJ software. Different types of granulomas (necrotic, multicentric or nascent) and disseminated bacteria were classified according to examples shown in Fig. S2A. Granulomas were counted manually from each section to determine the total number of granulomas in each fish (Oksanen et al., 2013).

Hypoxic lesions in granulomas were visualized with the HypoxyProbe-1 kit (HP1-100Kit, Hypoxyprobe, Burlington, MA, USA) in fish with a latent infection and after 3 weeks of reactivation with dexamethasone. Fish were anesthetized with 0.02% 3-aminobenzoic acid ethyl ester and intraperitoneally injected with pimonidazole hydrochloride (60 µg/fish), which forms covalent adducts with sulphydryl groups within protein structures under hypoxic conditions. The fish were transferred to fresh water for 5-10 min before euthanasia and histological sections were prepared as described above. The staining followed the manufacturer's instructions with some modifications. Glasses were first deparaffinized and boiled in a 10 mM sodium citrate buffer with 0.05% Tween (pH 9) at 98°C for 15 min to retrieve antigens. To block endogenous peroxidase activity, glasses were treated for 5 min with 3% hydrogen peroxidase (88597, Sigma-Aldrich) before a 1-h incubation with the primary antibody, a 1:600 dilution of Hyproxyprobe-1 Mab1 (Batch No. 5.23.15). The secondary antibody, universal immuno-enzyme polymer-peroxidase conjugate (mouse) (414131F, Nichirei Biosciences Inc., Tokyo, Japan), was incubated for 30 min. For the staining reaction, 15 µl/ml of the chromogen (ImmPACT DAP chromogen, Vector Laboratories, Burlingame, CA, USA) was mixed with ImmDACT™ DAB (Vector Laboratories), and incubated for 5 min. Mayer's Hematoxylin solution (Reagena, Toivala, Finland) was used as a counterstain for 2 min. TBS with 0.05% Tween buffer was used for all washing steps and the staining was performed with a Autostainer 480 machine (Lab Vision, Thermo Fisher Scientific). Finally, glasses were dehydrated within increasing alcohol series to xylene and embedded with Coverquick 2000 (VWR Chemicals, Leuven, Belgium), and scanned as described above. The total number of hypoxic and nonhypoxic granulomas was counted from both groups (*n*=2 fish/group).

### FACS

For the FACS analysis, wild-type AB fish and Tg:lck(*lck-EGFP*) fish were fed with dexamethasone-containing food for the indicated times, together with an untreated control group (12 fish/group); and latently infected and uninfected AB fish were treated with dexamethasone or normal fish food for 1 week. Fish were euthanized with 0.04% 3-aminobenzoic acid ethyl and their kidneys were dissected in 500 µl of 0.5% fetal bovine serum (Sigma-Aldrich) in PBS on ice. Tissues were homogenized and filtered through a 35 µm filter cap by centrifugation at 200 ***g*** for 1 min. Cell sorting was performed with a FACSCanto II device (Becton Dickinson Biosciences, San Jose, CA, USA) and with the FACSDiva software (Becton Dickinson Biosciences). The samples were run at medium speed (1000 events/s) and 20,000 events were recorded for each sample, and used for the analysis of different cell populations (lymphocytes, blood cell precursors, granulocytes and monocytes) based on GFP expression, granularity and size with FlowJo software.

### qPCR and qRT-PCR

Bacterial counts for each fish were obtained with qPCR (SensiFAST™ SYBR^®^ No-ROX kit, Biolane Reagents Ltd, UK). The contents of the intraperitoneal cavity from each fish was used for DNA extractions and the Tri-reagent was used for the isolations as described previously (Parikka et al., 2012). In qPCR, primers specific for the *M. marinum* internal transcribed spacer forward (5′-CACCACGAGAAACACTCCAA-3′) and reverse (5′-ACATCCCGAAACCAACAGAG-3′) were used. To exclude the possibility of a background *M. marinum* infection, a group of uninfected fish treated with dexamethasone was also subjected to the qPRC analysis.

For the expression analysis of cytokines and T cell markers, whole kidneys were dissected and used for RNA isolation with the RNeasy Mini Kit (Qiagen). RNA samples were purified with the RapidOut DNase Removal kit (Thermo Fischer Scientific) and then used for qRT-PCR (SensiFAST™ SYBR No-ROX Kit, Biolane Reagents Ltd), according to the manufacturer's instructions. Expression levels of cytokines were normalized to the expression level of *elongation factor 1-alpha* (*EFa1*; *eef1a1/1*) in each sample. The primers used are shown in Table 1. The qPCR was run with a Bio-Rad CFX96™ thermal cycler, and the data were analyzed with the Bio-Rad CFX96™ Real Time System software. Excluding the kidney, the rest of the internal organs were used for DNA extraction and determining the bacterial counts as above.

**Table 1.**
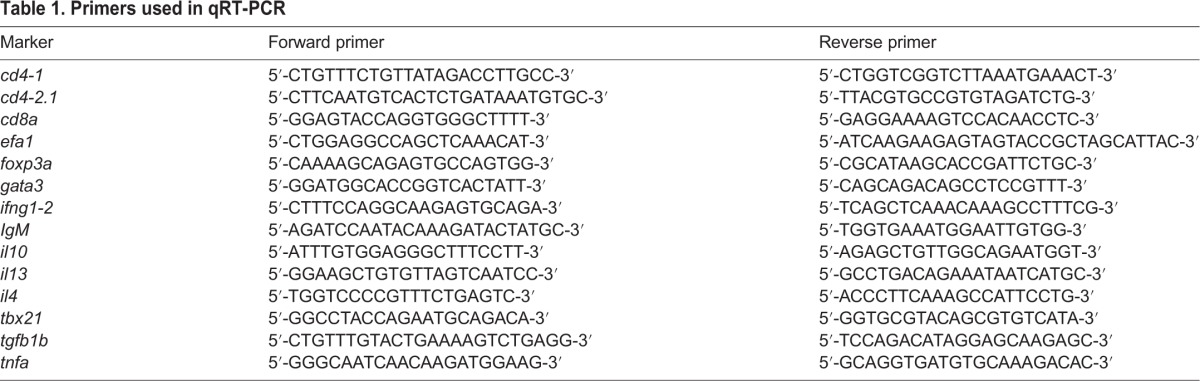
**Primers used in qRT-PCR**

### *In vitro* antibiotic testing with a bioluminescent *M. marinum*

To test the effectiveness of antimicrobial drugs *in vitro*, we exposed a bioluminescent *M. marinum* strain, ATCC BAA535, with the pMV306 plasmid (Addgene plasmid 26161), containing the LuxABDE cassette and kanamycin resistance (Andreu et al., 2010), to different concentrations of antibiotics (ethambutol E4630, metronidazole M3761, isoniazid I3377 and amikacin A0368000, Sigma-Aldrich) and measured the light emitted by the bacteria with a 2014 EnVision Multilabel Reader (PerkinElmer, Waltham, MA, USA). Antibiotic concentrations were selected based on the literature (de Steenwinkel et al., 2010). For each tested concentration, six replicate samples were prepared in a white 96-well plate; in each well the desired antibiotic dilution was added to 200 µl of a bacterial dilution (500 cfu/µl). Water was used as a negative control. Mycobacteria were incubated at 29°C for 7 days and the bioluminescence was measured on days 0, 1, 2, 3, 4 and 7. The results were analyzed with EnVision Workstation 1.12 (PerkinElmer).

### DNA vaccinations

DNA vaccine constructs were prepared as in former studies (Oksanen et al., 2013; Myllymäki et al., 2017). Briefly, antigens were cloned into plasmids (pCMV-*EGFP*, Addgene plasmid 11153) and transformed into chemically competent *E. coli* (One Shot TOP10 cells, Invitrogen)*.* Plasmid DNA was isolated with a Plasmid Plus Maxi Kit (Qiagen, Venlo, The Netherlands). Fish were infected with a low dose of mycobacteria, and 5 weeks postinfection vaccinated with 12 µg plasmid or a mixture of plasmids into the dorsal muscle, followed by electroporation to the injection site (six pulses of 40 V, 50 ms each) (Oksanen et al., 2013). The *EGFP* expression of vaccine constructs was detected in each fish under UV light 5-7 days postvaccination (Myllymäki et al., 2017). Five weeks postvaccination, dexamethasone feeding (10 µg/fish/day) was started lasting for 4 weeks, followed by determination of bacterial burden in each fish as described above. In the primary screen, 11-18 fish per group were immunized with the antigen candidates. Of these, the antigens leading to a significant reduction in bacterial counts (two-tailed Mann–Whitney test) were chosen for a replicate experiment with similar group sizes to confirm the original result.

### Statistical analyses and power calculations

Statistical analyses were carried out with GraphPad Prism 5 version 5.2 (Software Inc.). Bacterial burdens per fish were analyzed with the one- or two-tailed Mann–Whitney test, and the granuloma counts were analyzed with unpaired Student's *t*-test and Fisher's exact test of independence for hypoxic and nonhypoxic granulomas. Two-way ANOVA with a Bonferroni posttest was used for the statistical analysis of the cell populations in the FACS analysis, and the qRT-PCR results were analyzed with a two-tailed Mann–Whitney test. The effectiveness of the antibiotics *in vitro* was assessed with one-way ANOVA with Friedman's test. In each case, *P*<0.05 was considered significant. In the antibiotic and vaccine screening experiments, the sample size of each group was determined based on power calculations as in Myllymäki et al. (2017).

## Supplementary Material

Supplementary information

First Person interview
