# Supplementary material for: Identification of protective postexposure mycobacterial vaccine antigens using an immunosuppression-based reactivation model in the zebrafish
Source: Dis Model Mech. 2018 Mar 1;11(3):dmm033175. doi: 10.1242/dmm.033175 (PMC5897733; doi:10.1242/dmm.033175)
Supplement: Supplementary information [file dmm-11-033175-s1.pdf]

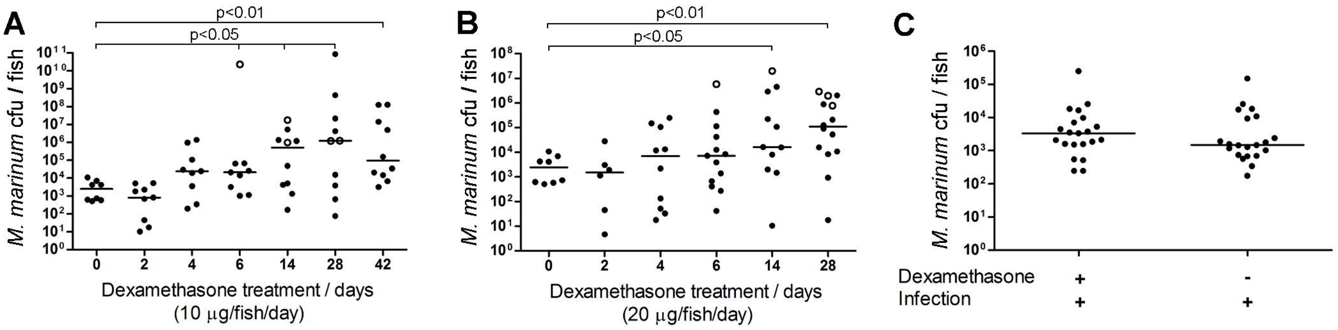

Supplementary Figure 1. Dexamethasone treatment increases bacterial loads in zebrafish with a latent mycobacterial infection.

(A) Zebrafish with a latent *M. marinum* infection were treated with dexamethasone 10 µg/fish/day (A) or 20 µg/fish/day (B) for indicated times. Each dot shows the bacterial count in one fish (n=8-14 fish/group). (C) The fish were treated with dexamethasone (10 µg/fish/day) for four weeks prior to a low dose *M. marinum* infection. The bacterial burden was quantified 4 weeks post infection. Horizontal lines represent the median bacterial count of each group. p values were calculated using the one-tailed Mann-Whitney test.

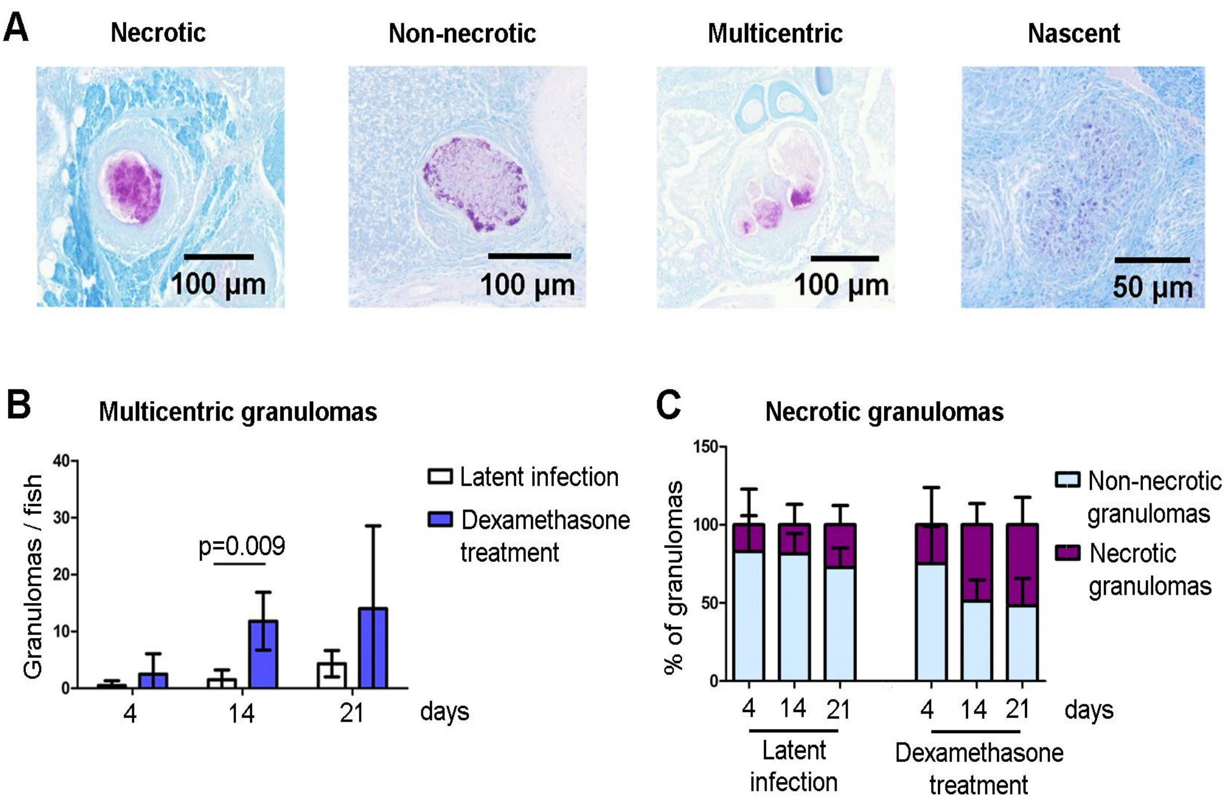

Supplementary Figure 2. Dexamethasone treatment increases the number of multicentric granulomas and the relative proportion of necrotic granulomas.

(A) Classification of different types of granulomas with Ziehl-Neelsen staining used for the quantification in Figure 2 and in this figure. (B) Quantification of multicentric granulomas per fish in a latent infection compared to a dexamethasone treatment. The bars shows mean±s.d. (n=3-6 fish/group). (C) The proportion of necrotic and non-necrotic granulomas in a latent infection and after dexamethasone treatment (n=3-6 fish/group). Bars show the mean percentage±s.d. p values are calculated with two-way ANOVA.

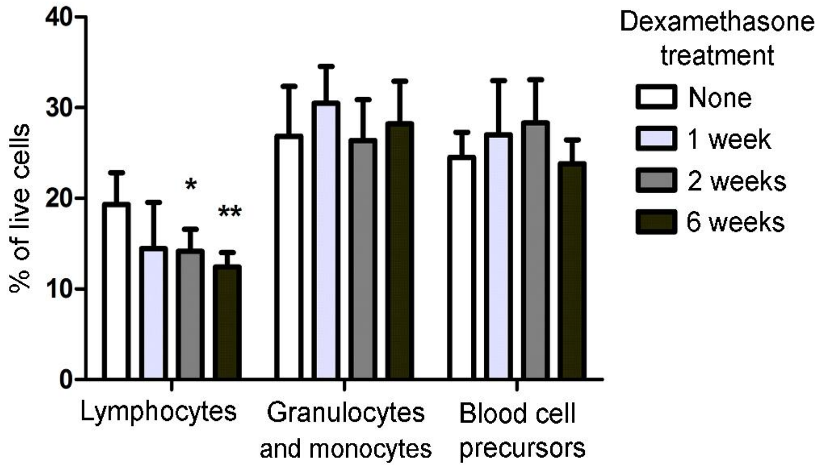

Supplementary Figure 3. Dexamethasone treatment decreases the relative amount of lymphocytes in the zebrafish kidney, but does not affect the granulocyte and monocyte or blood cell precursor populations.

Quantification of cell populations in the zebrafish kidney by FACS analysis following a dexamethasone treatment for 1, 2 and 6 weeks. n=7-9 fish/group. Data is presented as mean ±s.d. The data was analyzed with two-way ANOVA with Bonferroni's posttest, * $p<0.05$, ** $p<0.01$.

Disease Models & Mechanisms • Supplementary information

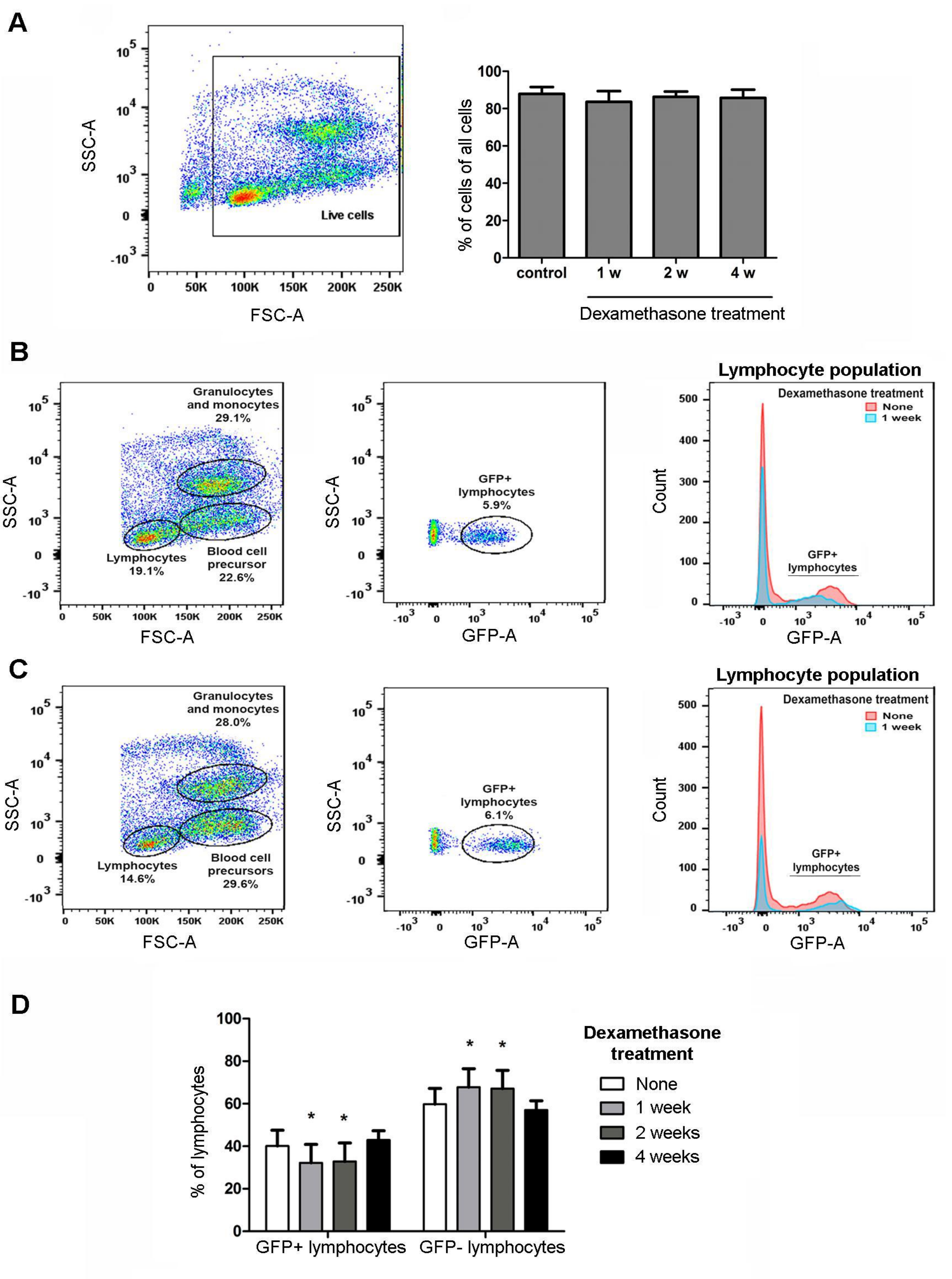

Supplementary Figure 4. Dexamethasone treatment does not affect the number of live cells in the zebrafish kidney or their gating, but has a mild effect on the proportion of GFP+ and GFP- lymphocytes.

A) The cells in the live gate were included in the FACS analysis and their number remained constant during a dexamethasone treatment. B and C) Gating of the kidney cells based on size (SSC-A) and granularity (FSC-A) and GFP expression after two (B) and four (C) weeks of a dexamethasone treatment. C) The relative proportion of GFP+ lymphocytes decreases during the first two weeks of a dexamethasone treatment, but is restored to the level of untreated fish by four weeks. n=12. Data is presented as mean±s.d. The data was analyzed with two-way ANOVA with Bonferroni's posttest, * $p<0.05$, ** $p<0.01$, *** $p<0.001$.

Disease Models & Mechanisms • Supplementary information

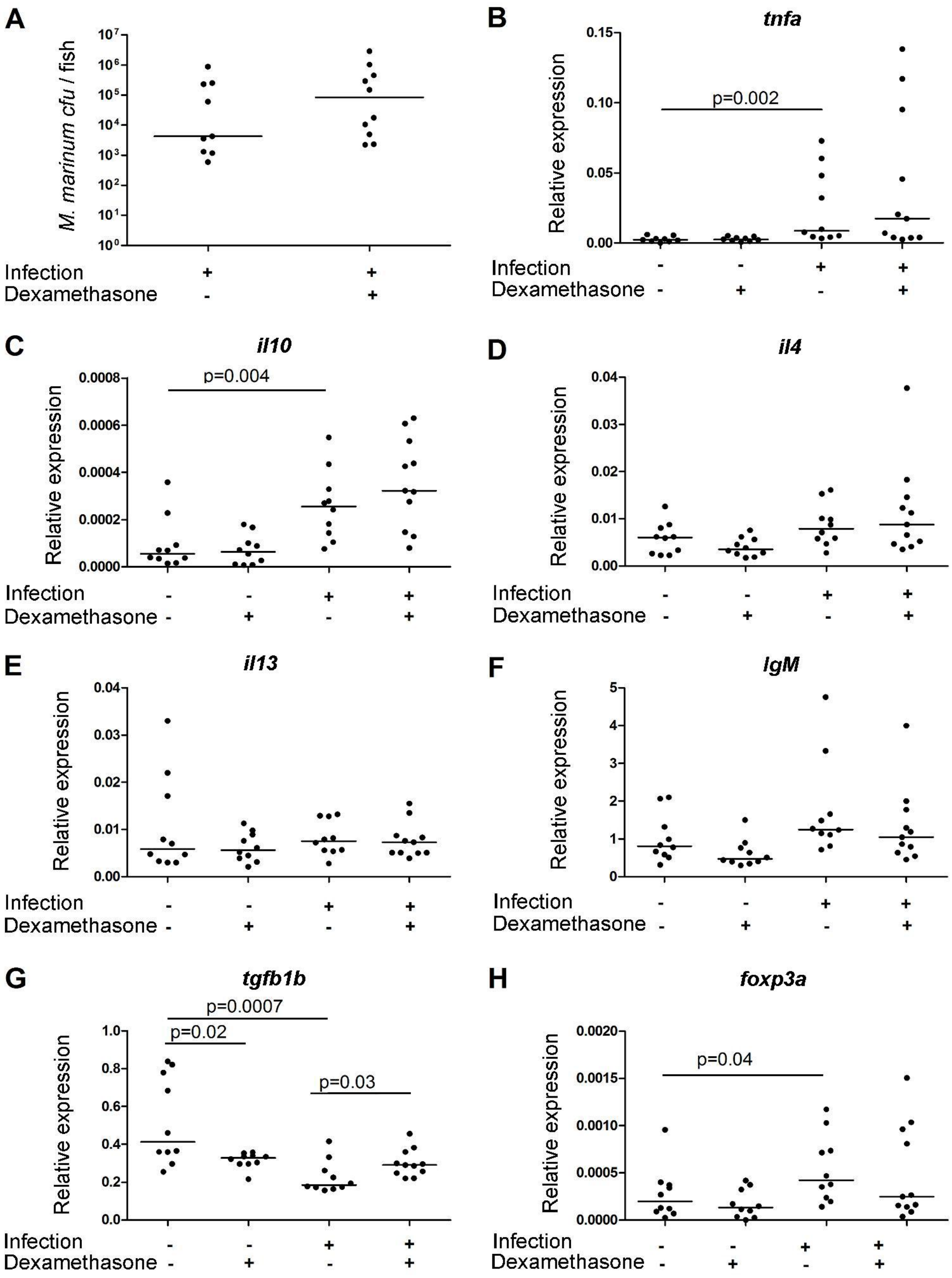

Supplementary figure 5. Mycobacterial infection increases the expression level of *tnfa* and *il10* and a dexamethasone treatment increases the expression of *tgfb1b* upon infection.

(A) The bacterial burden per fish in a latent infection and after a one-week dexamethasone treatment. Horizontal lines are median values of each group (n=9-10 fish/group). (B –H) Relative expression levels of cytokines and lymphocyte markers in zebrafish kidneys. Expression levels were normalized with the expression level of *ef1a*. Horizontal lines show median values of each group (n=9-10 fish/group). p values were calculated using the two-tailed Mann-Whitney test. (B) *tnfa*, (C) *il10*, (D) *il4*, (E) *il13*, (F) *IgM*, (G) *tgfb1b*, (H) *foxp3a*.

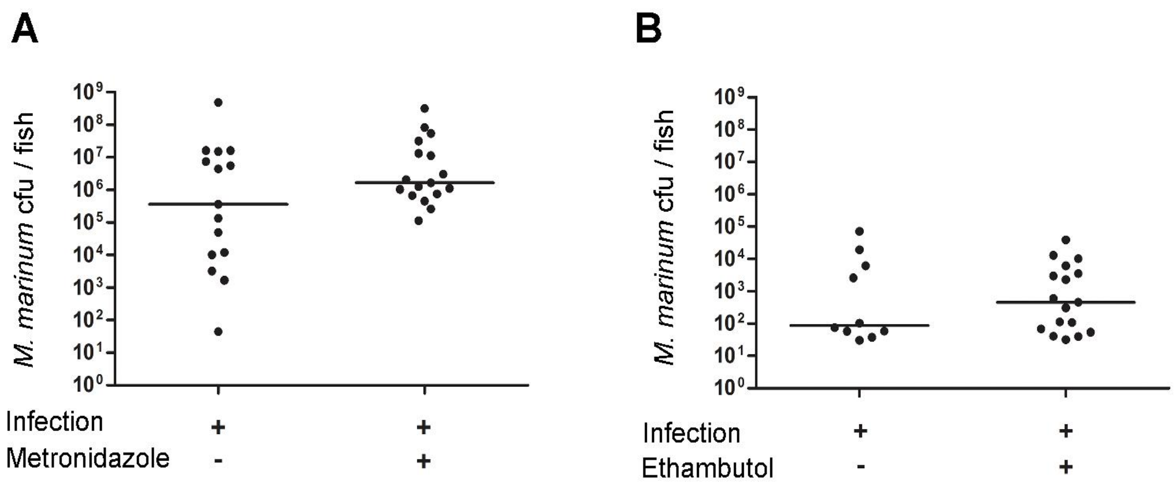

Supplementary Figure 6. Metronidazole and ethambutol treatment against a latent mycobacterial infection in adult zebrafish.

(A) Fish with a latent mycobacterial infection were treated three weeks with metronidazole (50 µg/fish/day), and (B) two weeks with ethambutol (50 µg/fish/day). Each dot represents the bacterial count in one fish. Horizontal lines show the median value of each group. n=10-15 fish/ group. The two-tailed Mann-Whitney test was used for the statistical analysis.
