# Supplementary material for: Identification of protective postexposure mycobacterial vaccine antigens using an immunosuppression-based reactivation model in the zebrafish
Source: Dis Model Mech. 2018 Mar 1;11(3):dmm033175. doi: 10.1242/dmm.033175 (PMC5897733; doi:10.1242/dmm.033175)
Supplement: First Person interview [file dmm-11-033175-s2.pdf]

© 2018. Published by The Company of Biologists Ltd | Disease Models & Mechanisms (2018): doi:10.1242/dmm.033175: Supplementary information

FIRST PERSON

First person – Henna Myllymäki and Mirja Niskanen

First Person is a series of interviews with the first authors of a selection of papers published in Disease Models & Mechanisms, helping early-career researchers promote themselves alongside their papers. Henna Myllymäki and Mirja Niskanen are joint first authors on 'Identification of protective postexposure mycobacterial vaccine antigens using an immunosuppression-based reactivation model in the zebrafish', published in DMM. Henna is a postdoctoral researcher and Mirja is a PhD student in the Experimental Immunology group led by Prof Mika Rämet at the University of Tampere, Finland, investigating tuberculosis using *Mycobacterium marinum* infection of zebrafish as a model.

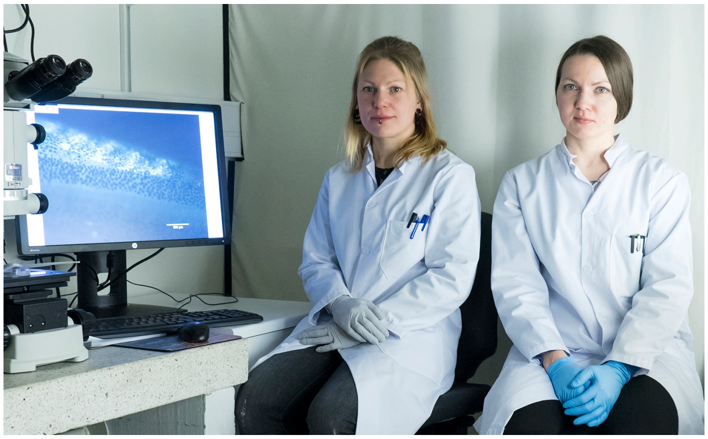

Henna Myllymäki (left) and Mirja Niskanen (right) at the fluorescent microscope, confirming the expression of GFP-tagged antigens in immunized fish.

How would you explain the main findings of your paper to non-scientific family and friends?

HM: Tuberculosis is still a major health problem in many areas, and it has been estimated that one third of the human population carries the bacterium *Mycobacterium tuberculosis* in a latent, quiescent form. A latent infection can reactivate into active tuberculosis even decades after the initial contact, and currently there are no means to prevent this. The risk of reactivation of tuberculosis is especially high in people who are immunocompromised, such as those who also have an HIV infection, take medication for arthritis or undergo chemotherapy. We model the reactivation of a latent TB infection in zebrafish using a bacterium that is a close relative of *M. tuberculosis* and by feeding the fish with an immunosuppressive medicine. We found that this led to the loss of certain types of immune cells, which indeed resembles the situation in HIV-infected people. We found out that a certain antibiotic or a few DNA vaccine candidates could protect the zebrafish against the reactivation of a mycobacterial infection. Therefore, we think this zebrafish model could be used for discovering medical solutions against the reactivation of TB, such as for pre-clinical screening of new vaccines.

MN: Usually, I need to simplify my study quite a lot when I am explaining it to my family or friends. I start by telling them that I am studying tuberculosis in a zebrafish model and our goal is find a new type of tuberculosis vaccine that would prevent the reactivation of latent tuberculosis. This is important, since one third of the human population is estimated to have this asymptomatic form of tuberculosis, which may reactivate to the active disease. In this particular study, we developed a method to reactivate latent infections with a medicine called dexamethasone. This chemical suppresses the immune system of fish, which enables the 'sleeping' mycobacteria to wake up and form an active, infective tuberculosis. We use this model to test the effectiveness of new vaccine candidates; if our experimental vaccine is effective, fish will not get ill even though they are exposed to a dexamethasone treatment.

"It would be great to see more and more researchers choosing a zebrafish model for their studies. This could also have a positive impact on the amount of zebrafish-specific tools available for research purposes."

What are the potential implications of these results for your field of research?

MN & HM: Even though tuberculosis is an ancient disease and has been studied extensively, we have limited means to treat patients with tuberculosis and prevent infections. It seems that *M. tuberculosis* is still a step ahead of researchers and we have a lot to investigate to overcome this battle between humans and mycobacteria. For instance, the exact reactivation mechanisms of latent tuberculosis remain elusive. Our study presents a dexamethasone-based reactivation model, which provides a tool to study mechanisms of reactivation and make observations from both the host and mycobacterial side. With this method, it is possible to characterize the mechanisms leading to reactivation and thus gain a better understanding of the process, which may be critical when new vaccines or antimicrobial chemicals against tuberculosis are developed.

We hope that this study also provides a convincing example of how feasible zebrafish is as a model organism. It would be great to see more and more researchers choosing a zebrafish model for their studies. This could also have a positive impact on the amount of zebrafish-specific tools available for research purposes.

What are the main advantages and drawbacks of the model system you have used as it relates to the disease you are investigating?

HM: A great advantage of the zebrafish model of tuberculosis is the ability to use a natural host–pathogen pair, and to do that using a pathogen that belongs to biosafety level (BSL)2, instead of BSL3 like *M. tuberculosis*. In addition, the adult zebrafish has a fully developed adaptive immune system and can be immunized against mycobacteriosis with BCG or DNA vaccines. Using the gelatin coating of food, the fish can be non-invasively subjected to different

Henna Myllymäki and Mirja Niskanen's contact details: BioMediTech Institute and Faculty of Medical and Life Sciences, University of Tampere, Tampere FI-33014, Finland.

E-mails: henna.myllymaki@staff.uta.fi; mirja.niskanen@uta.fi

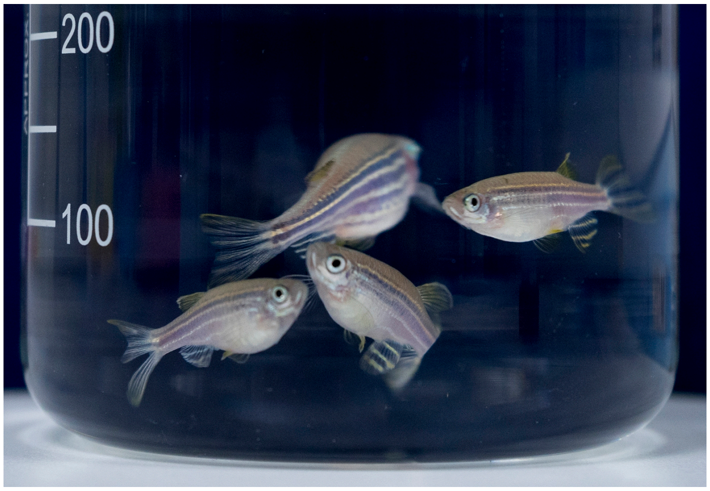

The adult zebrafish used in the *M. marinum* infection model. Photo credit: Lauri Paulamäki.

drugs to target mycobacteria or to manipulate the fish immune system. As for the dexamethasone treatment, an obvious advantage is that it is reversible and does not cause any apparent harm to the fish itself.

In zebrafish, there is still a lack of many immunological reagents and tools, such as antibodies or cell lines. Compared with the larval zebrafish model, a drawback with the adult zebrafish is the inability to monitor the progression of an infection in real time, since the fish are no longer transparent, nor can you take samples of, for example, blood. This would be especially useful since often it takes a long time to get to the end-point analysis. In addition, there is relatively little information on the functions of mycobacterial genes, especially concerning *M. marinum*.

MN: In our study, we use the zebrafish-*M. marinum* infection model. In the lab environment, zebrafish is a good animal to study tuberculosis. *M. marinum* is natural pathogen of fish and causes a similar disease in zebrafish to *M. tuberculosis* in humans. There are both active and naturally latent phases of infections, which are not seen in most experimental animal models. For immunological studies, zebrafish is a suitable model since it has both innate and adaptive immunity. Zebrafish immunity appears to have similar features to humans, such as immune cells and cytokines. The main drawback of zebrafish is the anatomical differences, especially the lack of lungs. However, the small size and relatively low housing costs make zebrafish a feasible model for screening studies.

What has surprised you the most while conducting your research?

HM: Since dexamethasone causes developmental defects in zebrafish larvae, we were slightly worried that it could cause dramatic alterations in immune cell populations and affect the expression of various genes in the adult zebrafish too, which would make characterization of the reactivation process with this model system very difficult. Therefore, it was an encouraging surprise to see that in fact, dexamethasone treatment only affected the lymphocyte population, and only a limited number of genes within those cells. In addition, this seems to resemble the situation in HIV–TB-coinfected humans, suggesting the system could be used for investigating therapeutic solutions for these people as well.

MN: As a young scientist, I feel like Alice in Wonderland and many things in the lab have surprised me. I am still amazed how nicely the zebrafish-*M. marinum* infection model resembles human tuberculosis. The granuloma structures, which we have detected with different histological staining methods, are especially fascinating!

Describe what you think is the most significant challenge impacting your research at this time and how will this be addressed over the next 10 years?

HM: A major challenge in searching for a way to prevent or cure tuberculosis is that we do not really know what we are looking for. That is, there is no definition of what kind of immune response could prevent a tuberculosis infection, eradicate an existing infection or prevent its reactivation. Also, we could use a more comprehensive understanding of the bacterial life cycle, including its various immune evasion strategies. Of course, there are data concerning the immune responses and markers associated with different outcomes of tuberculosis, coming from both humans and different model organisms. With the help of modern sequencing methods, I think that in the next decade all this data can be better utilized to form a more uniform picture of the protective immune response against tuberculosis. This should aid both the development of new drugs and vaccines, and the use of different model organisms in designing and testing them.

What changes do you think could improve the professional lives of early-career scientists?

MN: In my opinion, many things are well organized in our university. We have lots of peer support, colloquiums, seminars, doctoral school studies, follow-up groups and possibilities to take part in international conferences. However, it could be beneficial to have some kind of mentoring program. Mentors could give support throughout the project, encourage setting further goals and thinking about your career in a broader sense. This could increase the self-confidence and motivation of early-career scientists.

What's next for you?

HM: In the current paper, we have studied the reactivation of a latent mycobacterial infection, focusing on the host's side of the story. The next thing would be to elucidate the bacterium's side of the story; for example, by studying what exactly makes a bacterium 'reactivate', and how this differs from dormancy at the cellular and molecular levels. In addition, now that we have the zebrafish reactivation model set up and validated, we will continue with vaccination studies.

MN: In terms of this study, the next step is to identify and characterize mycobacterial genes that are expressed during reactivation, and to test those as possible vaccine antigens. For me personally, the next goal is to complete my PhD thesis.

Reference

Myllymäki, H., Niskanen, M., Luukinen, H., Parikka, M. and Rämet, M. (2018). Identification of protective postexposure mycobacterial vaccine antigens using an immunosuppression-based reactivation model in the zebrafish. *Dis. Model. Mech.* **11**: dmm033175.

Disease Models & Mechanisms
